# Collective effects of human genomic variation on microbiome function

**DOI:** 10.1038/s41598-022-07632-3

**Published:** 2022-03-09

**Authors:** Felicia N. New, Benjamin R. Baer, Andrew G. Clark, Martin T. Wells, Ilana L. Brito

**Affiliations:** 1 Meinig School of Biomedical Engineering, Cornell University, Ithaca, NY USA; 2 Department of Statistics and Data Science, Cornell University, Ithaca, NY USA; 3 Department of Computational Biology, Cornell University, Ithaca, NY USA

**Keywords:** Computational models, Microbiota, Genetics research

## Abstract

Studies of the impact of host genetics on gut microbiome composition have mainly focused on the impact of individual single nucleotide polymorphisms (SNPs) on gut microbiome composition, without considering their collective impact or the specific functions of the microbiome. To assess the aggregate role of human genetics on the gut microbiome composition and function, we apply sparse canonical correlation analysis (sCCA), a flexible, multivariate data integration method. A critical attribute of metagenome data is its sparsity, and here we propose application of a Tweedie distribution to accommodate this. We use the TwinsUK cohort to analyze the gut microbiomes and human variants of 250 individuals. Sparse CCA, or sCCA, identified SNPs in microbiome-associated metabolic traits (BMI, blood pressure) and microbiome-associated disorders (type 2 diabetes, some neurological disorders) and certain cancers. Both common and rare microbial functions such as secretion system proteins or antibiotic resistance were found to be associated with host genetics. sCCA applied to microbial species abundances found known associations such as *Bifidobacteria* species, as well as novel associations. Despite our small sample size, our method can identify not only previously known associations, but novel ones as well. Overall, we present a new and flexible framework for examining host-microbiome genetic interactions, and we provide a new dimension to the current debate around the role of human genetics on the gut microbiome.

## Introduction

Variation in gut microbiome composition underlies numerous human phenotypes and health outcomes, such as immune function, metabolic disorder, cancer and psychiatric traits. These variations have been attributed largely to environmental factors, including diet^1^, antibiotic exposure^2^, and birth modality^3^. However, twin and population-based genetics studies have identified genetic associations with the overall composition of the gut microbiota^4–12^, in addition to heritable taxa^13,14^. Underpowered studies, population differences, as well as inconsistencies in experimental and computational methods, have led to a problem of replicability across microbiome genome-wide association studies (GWAS) and contribute to the doubt surrounding the role of genetics in shaping the gut microbiome^1,15^.

Traditional methods to associate host genetics with microbiome traits involve comparing a single genotype to a single microbial species or pathway (one-vs-one) or a single genotype to a set of microbial species (one-vs-many association tests, Spearman’s rank correlation)^4,6–13^. As the number of species within a microbiome can be several orders of magnitude greater than the sample size, there is limited statistical power and a risk of overfitting. Until the cost and computational burden of microbiome profiling decreases, it will be difficult to reach the sample sizes of modern human GWAS, which now reach into the hundreds of thousands of participants even for single phenotypes^16^. The sample size needed to perform reasonably efficient inference can be reduced by performing association tests between one genotype and the dissimilarity between the study’s microbiome samples, which reduces the dimension of multivariate phenotypes such as the gut microbiome^1,13^. Although the microbiota function as a community, these gross metrics (e.g., beta diversity) obscure the sources and mechanisms of the host-microbe genetic relationship and their effect sizes tend to be small. Furthermore, similar to the observation that human GWAS studies focusing on single SNP-level associations do not account for the total observed heritability^17^, microbiome GWAS studies may not account for vertical- and family-level heritability of microbiome components^18,19^.

Since microbial genomes are not static due to recombination between closely related strains and horizontal gene transfer^20–22^, species-level analyses can introduce avoidable variability in comparing results across studies. Metagenomic sequencing offers an opportunity to examine microbial genes, which offers a more consistent accounting of a microbiome’s functional capacity across individuals and may capture more specific mechanisms underlying various phenotypes. Not surprisingly, GWA studies using metagenomes are few, as the cost of metagenomic data acquisition can be roughly tenfold higher than 16S rRNA profiles. Furthermore, the number of genes within a metagenome far outweighs the number of species, exacerbating the problem of dimensionality. Aggregating genes into functional units such as protein families, KEGG orthologs, or pathways reduces the dimensionality of the data, though these groupings can still outnumber sample sizes. Another challenge with metagenomic data is that they are right-skewed and are characterized by both zero-inflation and overdispersion. To appropriately model these data, we propose the use of a Tweedie distribution. Tweedie distributions are exponential dispersion families of distributions and are often used in generalized linear models^23^. Depending on their parameterization, they can have mass at zero along with non-negative continuous support. Tweedie distributions can also describe the mean to variance power law relationship that is present in several types of data including metagenomic abundances. These two characteristics have made it very useful in fields as diverse as ecology and natural language processing^24–27^. In ecology the distribution is commonly characterized through Taylor’s law.

**Figure 1:**
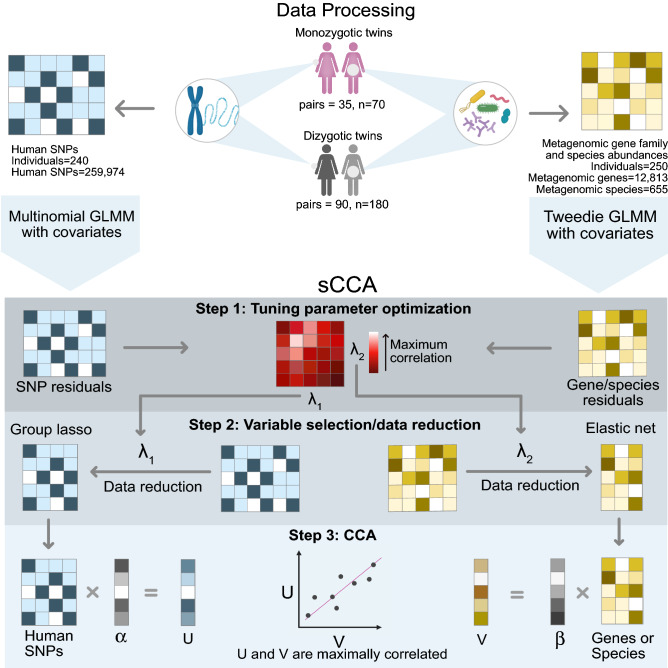
Overview of the sCCA method applied to the TwinsUK cohort. Data from the TwinsUK cohort include shotgun metagenome sequences and human variants from monozygotic and dizygotic female twins. Microbiome gene abundances or species abundances estimated from shotgun metagenomic sequencing, and host genotype data were input into generalized linear mixed models to extract residuals for downstream analysis (see Methods for details). Step 1 involves the tuning parameter optimization for sCCA. The pair of regularization parameters that represent the maximum correlation in the data are chosen for Step 2: Variable selection. In Step 2, we apply an elastic net to the sets of genes or species abundances and a group lasso to the human genotype data to reduce the size of the datasets. In Step 3, CCA is applied to the reduced data to find the maximally correlated linear combinations of the data. Figure drawn in Affinity Design (v1.10.4, https://affinity.serif.com/en-us/).

To address the challenge of identifying associations between human genetics and the composition and function of the gut microbiome, we apply a flexible, unsupervised, multivariate data integration method, known as canonical correlation analysis (CCA)^28^. This method bypasses the need for multiple hypothesis testing and leverages the combination of many small effect sizes. CCA is the earliest multi-table analysis method, first used in 1936 to relate multidimensional variables^28^. CCA creates low-dimensional representations of features, similarly to principal component analysis (PCA). However, unlike PCA, it allows for comparisons across multiple measurements, where the low dimensional representations of each set of features, or canonical components, represent the maximum correlation between the two sets of linear combinations. This method works well when the number of samples exceeds the number of measured features, which is not the case with modern genomics data. As an example, for a set of individuals, it is possible to correlate their RNA-seq gene expression data with their DNA copy number or SNP data. In this case, the number of genes with expression data and the number of polymorphisms will most likely exceed the number of individuals that can feasibly be sampled. Penalized CCA methods have been developed to overcome this issue^29–31^. In penalized CCA, also known as sparse CCA or sCCA, sparsity is induced in the features through penalization, for example using an *l*_1_ penalty (lasso)^32^. This method is flexible in two ways: (1) it can be applied to any number of measurements sampled from the same individuals or units, and (2) it can handle different penalization schemes, and 3) it does not require summarization of tables as preprocessing, unlike the beta diversity example. Here, we apply this method to human genotypes and corresponding metagenomic features from a set of 125 twin pairs that are part of the TwinsUK project^33^. Using sCCA and Tweedie distributions, we model the relationship between human SNPs and microbial species or gene family profiles, using appropriate penalties for each (Fig. 1).

## Results

### Study cohort and metagenomic processing

The TwinsUK project includes corresponding human genotypes and microbiome metagenomic shotgun sequences of 250 individuals. This dataset consists of female twin pairs from the United Kingdom, of which 35 are monozygotic and 92 are dizygotic^33,34^. A subset of 240 individuals were previously genotyped^13^. The SNP data were filtered to remove missing data, rare alleles (present in fewer than 10% of individuals), and loci violating HWE or in high linkage disequilibrium (> 80%) with another. After standard quality filtering, metagenomic sequences were assembled into contigs. We generated a custom microbial gene catalog from the metagenomic assemblies, totaling 5,025,174 microbial genes. These were combined into 12,813 annotated gene families present in at least 10% of the samples. Genes that could not be annotated with a KEGG functional group are excluded from the analysis. We also estimated species abundances from the metagenomes and found that 655 species are found in at least 10% of the samples.

Metagenomic shotgun sequencing data are compositional, which arises due to sequencing DNA from each sample that is equal to the library size, and proportional to the community size within each sample, and therefore results in relative abundance data of community members rather than absolute abundances. Such composition-based data imposes strong constraints on the correlations in relative abundances, and subsequent analysis must be done with caution. Mishandling of compositionality can make most results uninterpretable^35^. Microbiome 16S rRNA sequencing analyses have used rarefaction or sub-sampling techniques to mitigate the effects of compositionality, but this can result in the loss of data and can result in false positives^36^. Normalization methods for read counts assigned to a genomic feature, such as those used in RNA sequencing analyses, account for the number of reads sequenced per sample and other technical variability^37^, however, normalized abundances are still relative values^38^. Rather, by modifying a commonly used normalization method, RPKM (reads per kilobase sequenced per million)^37^, we include a term for the geometric mean of each sample’s abundances, thereby accounting for the compositionality of the data as well as the library size of each sample^39^. Our modified RPKM approach is a hybrid between Aitchison’s center log ratio transformation (CLR)^39,40^ and the standard RPKM approach. Rather than divide the number of mapped reads or the number of reads sequenced, we divide by the geometric mean (without taking the log) of the sample, thus adjusting for the length of the gene and the magnitude of the library in one step. Methods that address compositionality outperformed those that used relative abundances in a recent benchmarking study for various data transformation methods for microbiome studies^41^.

### Tweedie distribution for metagenomic abundance data

**Figure 2:**
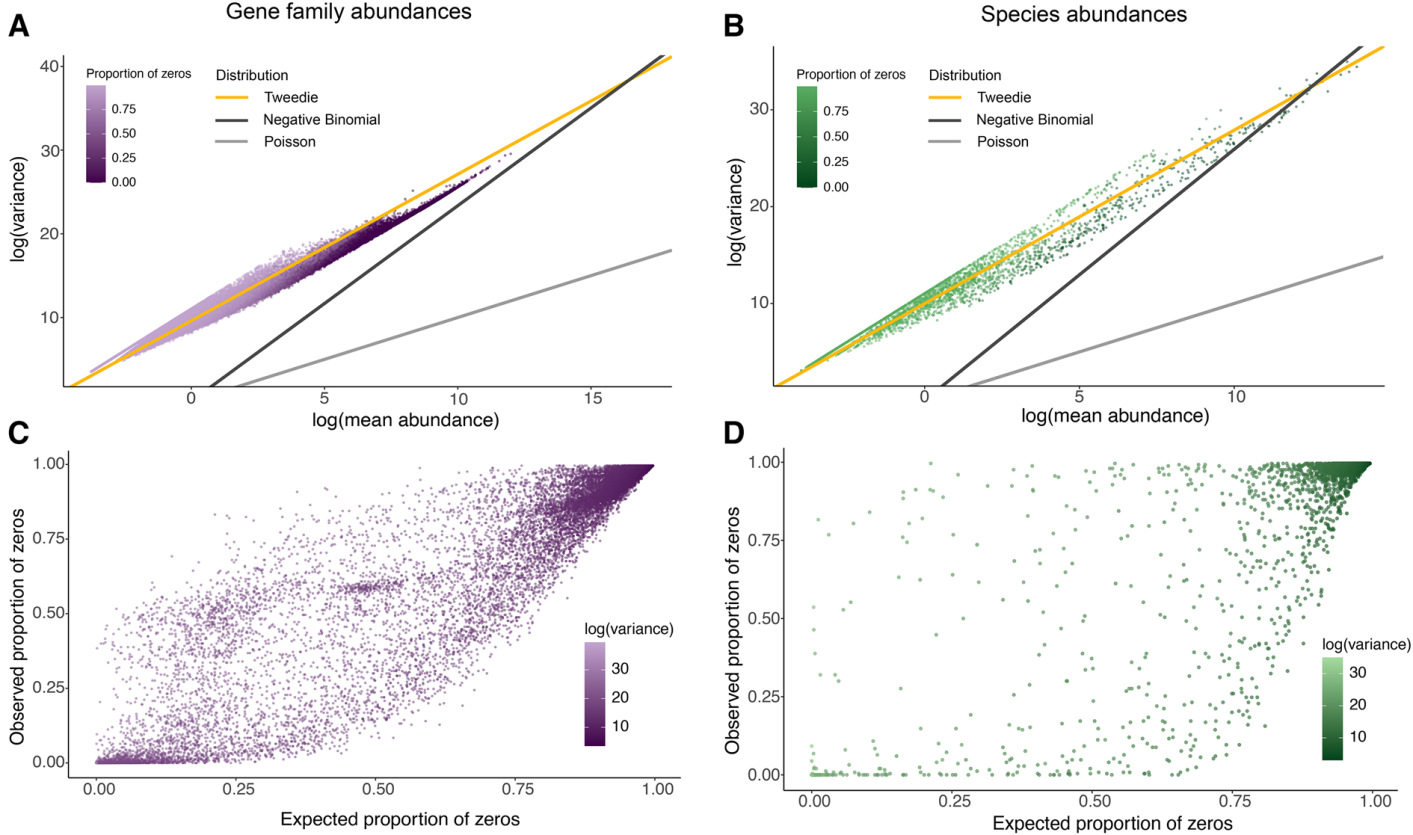
Tweedie distribution captures the mean-to-variance power structure of metagenomic abundance data. (**A**,**B**) the relationship between the log mean and log variance of the microbiome gene family abundances (**A**) and microbiome species abundances (**B**) from the TwinsUK 250 metagenomes. The yellow line represents the Tweedie distribution that best captures the mean to variance relationship, while the Poisson and negative binomial do not. (**C**,**D**) show the expected proportion of zeros per microbial gene family or microbiome species plotted against the observed proportion of zeros for the gene family abundances (**C**) and species abundances (**D**). The expected proportion is calculated from the Tweedie distribution where a linear (diagonal) relationship is expected. Figure created in R v4.1.2 (https://cran.r-project.org)^44^.

Normalized abundances from metagenomic sequencing are also right-skewed, overdispersed, and tend to be zero-inflated. The negative binomial and Poisson distributions are typically used to model sequencing data including RNA-seq and shotgun metagenomic sequencing^42,43^. However, the mean and variance of the Poisson distribution are the same. The negative binomial has a more flexible dispersion parameter than the Poisson, but it’s still not flexible enough: when comparing the log variance and the log mean, its intercept is always at *0* like the Poisson distribution (Fig. 2A,B). To account for zero-inflation and overdispersion within the normalized metagenomic counts, we introduce the Tweedie distribution to model the taxa and gene abundances from metagenomic shotgun sequencing. This is in contrast to some genomic methods which use zero-inflated Poisson and negative binomial distributions, which add an additional parameter and make them no longer exponential dispersion families. Metagenomic abundance data have a large mass at *0* and a long, positive tail, similar to the probability distribution for certain Tweedie distributions. The shape of a Tweedie distribution is determined by the shape parameter, *p*. When *1* < *p* < *2*, the distribution is continuous for *Y* > *0*, with a positive mass at *Y* = *0*. When *p* > *2*, the distributions are continuous for *Y* > *0*, without the mass at zero, and are no longer appropriate for zero-inflated data. The variance of the response variable is related to the mean through the Tweedie power and dispersion parameters, *p* and $\phi$, where $Var\left(Y\right)=\phi {\mu }^{p}$. We show that when *1* < *p* < *2*, the Tweedie distribution is flexible enough to capture the mean-to-variance power relationship in the metagenomic taxa and gene abundances (Fig. 2A,B)^23^. This Tweedie distribution captures the relationship better than either the negative binomial or Poisson, but it also accounts for the number of zeros present in the data. The expected number of zeros, given the count data, roughly matches the observed number of zeros specified by the Tweedie relationship, modeled as ${\text{P}}\left( {{\text{Y}} = 0} \right) = {\text{exp}}\left( {\frac{{ - \mu^{2 - p} }}{{\phi \left( {2 - p} \right)}}} \right)$, where $\phi$ is the dispersion parameter and *p* is the power parameter. For fixed $\phi$ and *p*, the probability of a gene count within a sample being zero decreases as the mean increases linearly (Fig. 2C,D).

### Sparse CCA identifies novel associations of host genetics with the gut microbiome

We use a sparse variant of CCA to identify associations between human genetics and features of the gut microbiome (Fig. 1). First, we identify appropriate penalization methods for each data type. To the human SNPs, which we have dummy-coded for additivity and dominance, we apply a group lasso^45^, and to the microbial species and gene family abundances we apply an elastic net^46^. Group lasso is used to select the entire SNP (since it is coded into columns), but with a different coding scheme it would be possible to use another method. Elastic net is useful for variable selection with genes and pathways because it tends to select correlated groups of variables, rather than selecting one at random from a group^46^. We apply a general linear mixed model to the SNPs and the microbial gene family and species abundances to control for the effects of sample shipment number, age at sampling, zygosity and family, and BMI. We include a random effect for the twin’s zygosity (monozygotic or dizygotic) to remove the effect of a person’s correlation with their own twin. This step allows the method to be generalizable to other cohorts since we are treating the individuals as if they were unrelated. The residuals are used as inputs to the sCCA pipeline. The first step in sCCA is tuning parameter optimization for the group lasso and elastic net. Next, using these optimized parameters for variable selection, sCCA is applied (Fig. 1). The output is the first canonical component, comprising a list of SNPs and metagenomic gene families or species, each with a corresponding weight. Features with non-zero weights are associated with each other across tables. To reduce variance and avoid overinterpretation, we ignore the value of the weights and only interpret weights as being zero or non-zero. To discover additional relationships using sCCA, we consider the part of the data unrelated to the previously found components in a process known as matrix deflation (see algorithm 2 of Witten et al.^30^). Matrix deflation considers the part of the SNPs and the gene abundances which cannot be explained by already recovered components.

To determine how stable the results of our sCCA method are, we reran the analysis using permuted versions of the data (the microbial function abundances, SNPs, and metadata). We created two permuted sets of the datasets and compared their results with the results from our real data. The results for the first permuted dataset overlapped less than 10% of the results, with 16/180 SNPs and 8/160 microbial functions. The results for the second permuted dataset overlapped with the original results with 9/170 microbial functions and 3/200 SNPs. The results of the two permuted datasets overlapped by 11 microbial functions and 13 SNPs. Given the small overlap between results, we expect a small number of false positives in our results.

To determine whether the same human SNPs associate with both species and bacterial gene families, we performed sCCA four times: one set of analyses on the species abundances and one set on the gene family abundances. For each of these two sets, we extracted two canonical components, for a total of four lists of human SNPs associated with either species or gene abundances from the gut microbiome. See Supplemental Table S1 for full sCCA results.

The first component of the sCCA test with microbial gene families identified 161 human SNPs and the second identified 171 human SNPs collectively associated with microbial gene family abundances. The analysis of the species abundances identified a similar number: 141 and 181 human SNPs that are associated with species abundances within the first and second component, respectively. We expect some level of overlap between the gene family and species tests, as it is likely that host genetics are associated with both for some species that contain certain genes. The overlap between components within a single analysis is also not surprising, because host genetics, or SNPs, could be associated with more than one dimension of the data. We find limited overlap between previous microbiome GWAS studies and our results at the level of SNPs. Twelve SNPs were found in common with previous studies: (rs9327097 [located within TNFAIP8], rs11578436, rs193466 [RARS1], rs60701 [DAP], rs6947185 [COL26A1], rs7638704, and rs1882926 [PDC-AS1]) from Kurilshikov et al.^12^ and rs906351 from Davenport et al.^11^. This is expected given the differences in methodologies.

**Figure 3:**
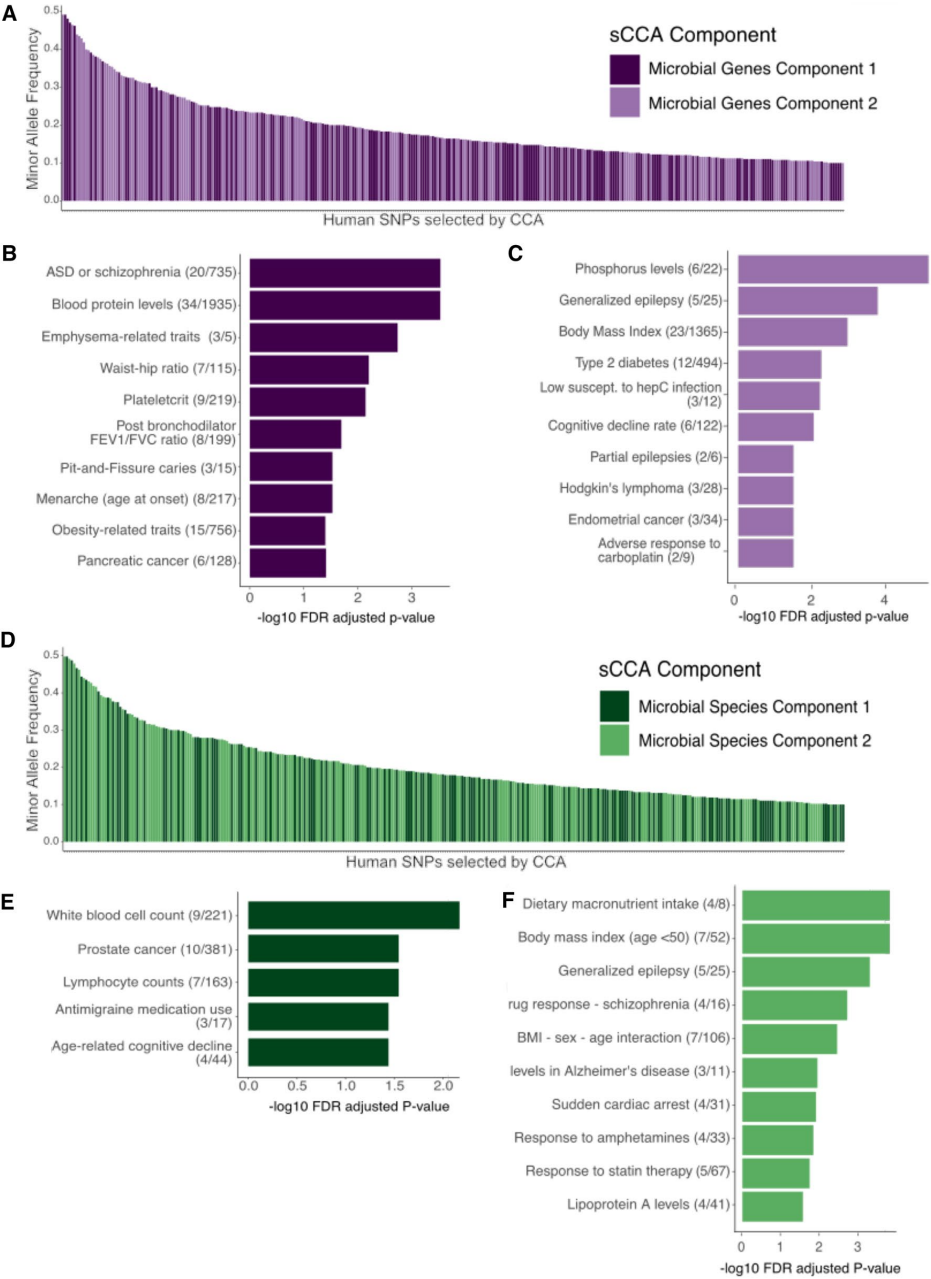
Human SNPs selected by sCCA as correlated with the gut microbiome from each analysis. (**A**–**C**) results from the correlation test of host genetics with the microbial gene family abundances of the gut microbiome, and (**D**–**F**) results from the test of host genetics with microbiome species abundances. (**A**,**D**) show the prevalence of the selected SNPs in the TwinsUK population, colored by which CCA component they were selected in (**B**,**C**,**E**,**F**) show the annotation of the human SNPs and their associated human genes that are associated with the gut microbiome genes and gut microbiota, respectively, the proportion of genes associated with our SNPs out of the total number of genes in each gene set in parentheses. For example, in (**B**) of the SNPs selected by component 1 that are associated with microbial genes, some are linked to 20 genes that are annotated as being associated with ASD or schizophrenia. Each bar plot shows the top 10 most significantly enriched traits for each test, Fisher’s exact test, FDR corrected − log_10_ *p* values. The entire list of significantly enriched traits can be found in Supplementary Table S3. Figure created in R v4.1.2 (https://cran.r-project.org)^44^.

We annotated the selected SNPs by their gene-disease associations and functions (GO terms) and performed enrichment analyses in FUMA GWAS^47^ (Fig. 3, for SNP enrichment results, see Supplemental Table S2). We see similarities between the many significantly enriched traits (Fisher’s exact test, FDR adjusted *p* value < 0.1) of our bacterial species and bacterial pathway sCCA analyses, which is also reflected in a small number of shared SNPs between the two analyses (53 SNPs overlapped in the first component of our bacterial species and bacterial genes sCCA analyses; and 12 overlapped in the second components of both analyses.) In both analyses, we see overlap of metabolic traits and diseases (obesity, BMI, blood pressure, type 2 diabetes), neurological diseases (Alzheimer’s disease, Schizophrenia, epilepsy, age-related cognitive decline), and cancer (pancreatic cancer, endometrial cancer, adverse response to chemotherapy, prostate cancer, Hodgkin’s lymphoma). Many of these traits reflect known links between the microbiome and metabolic disorders^48^, cancers^49^, and psychiatric disorders^50,51^.

### Microbial gene family CCA results

**Figure 4:**
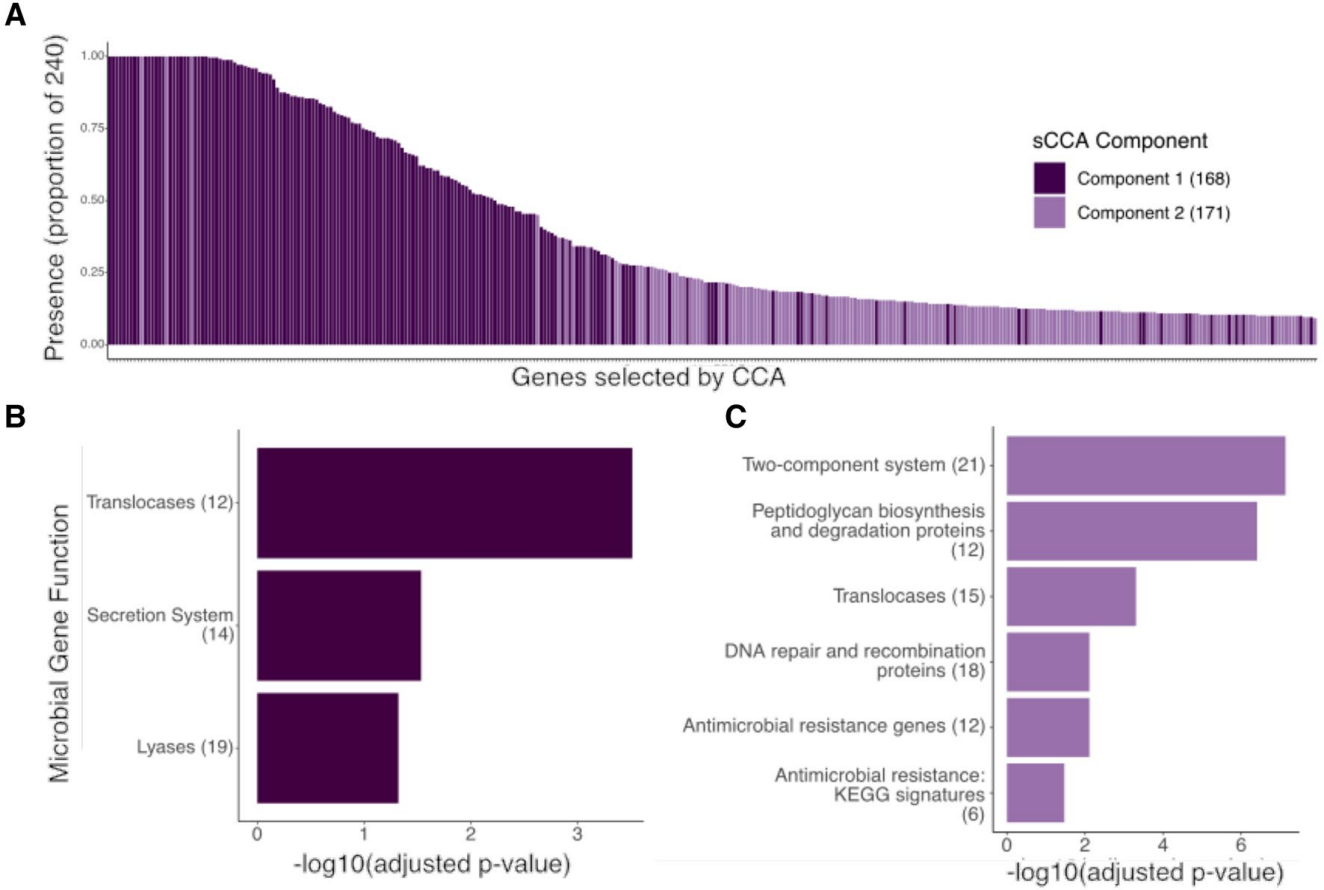
Microbial gene results. (**A**) the prevalence of the microbial genes selected by sCCA in the TwinsUK cohort (presence out of 240 individuals) colored by sCCA component. (**B**,**C**) the statistically significant functional enrichment of the microbial pathways, Fisher’s exact test, FDR corrected − log_10_ *p* values, in component 1 (**B**) and component 2 (**C**) with the number of genes in each pathway in parentheses. Microbial gene families that were selected by sCCA were grouped into KEGG module pathways, and enrichment tests were performed using all the pathways present in the samples as the background. Figure created in R v4.1.2 (https://cran.r-project.org)^44^.

We hypothesized that human genetics may have associations that span species and reflect specific functional gene families. From our two analyses on microbial gene family abundances, we find that 168 (1.31%) and 171 (1.33%) microbial gene families are associated with human genetics. Any two components of sCCA extract orthogonal information from the data. Here, this is reflected prominently in the abundances of the gene families between components 1 and 2. Component 1 selected more prevalent genes and is enriched for common enzymes (Fisher’s exact test, FDR *p* value < 0.1) (Fig. 4A). Conversely, component 2 selects rare genes (generally present in fewer than 50% of the population) and is enriched for rarer functions such as antimicrobial resistance (Fig. 4C). The first component’s results include functions with enrichment for secretion system and enzymes (Fisher’s exact test, FDR p < 0.1) (Fig. 4B). The second component is enriched for enzymes, two-component system, peptidoglycan biosynthesis and degradation proteins, DNA repair and recombination proteins, and antimicrobial resistance (Fisher’s exact test, FDR p < 0.1) (Fig. 4C). To further investigate the KEGG BRITE module known as “enzymes”, we performed an enrichment test of the subcategories of enzymes within the results. Within component 1, the enriched enzymes are translocases and lyases, and translocases are enriched in component 2 (Fig. 4B,C).

### Microbial species CCA results

**Figure 5:**
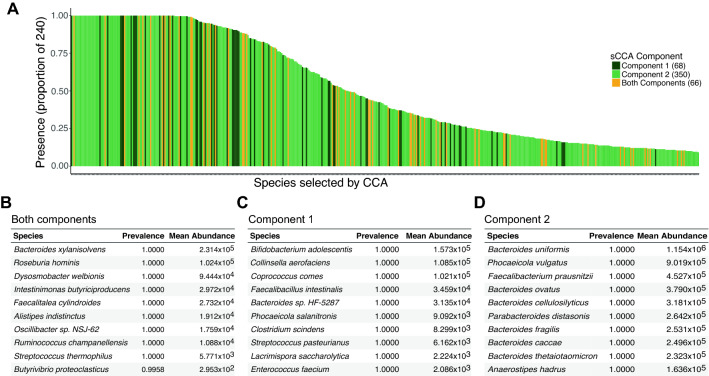
The prevalence of microbial species selected by CCA as being associated with host genetics. (**A**) Prevalence of the species out of 240 individuals. Data from components 1 and 2 are shown, with 66 species selected by both components. (**B**–**D**) The top 10 most prevalent species sorted by mean abundance selected by components (**B**), only component 1 (**C**), or only component 2 (**D**) that are associated with host genetics. Figure created in R v4.1.2 (https://cran.r-project.org)^44^.

When we apply this method to investigate microbial taxa abundances, we find 134 microbial taxa (20.45%) associated in the first component, and 417 microbial taxa (63.66%) associated in the second component from sCCA. Sixty-six of the detected species representatives overlapped between the two components, so in total, the abundances of 485, or 74.04%, of species in this analysis are associated with host genetics. Most of the species reflect organisms that are well-established with the commensal gut microbiome, although our analysis did detect associations involving potential pathogens such as *Phocaeicola vulgatus* (Fig. 5A–D). Similar to previous studies, we find that *Faecalibacterium prauznitsii* is associated with host genetics^6,52^, but we also find phage known to infect this species including Taranis, Toutatis, Lugh, Epona, and Oengus are associated with host genetics. We also identify crAssphage, uncultured crAssphage, and crAss001. The phageome is known to be unique to individuals and consistent over time with some evidence for a core gut phageome^53^. Several other species overlap with previous microbiome GWAS including the genera Streptococcus the genera *Barnesiella, Campylobacteracea, Lactococcus,* and *Akkermansia*^11^, *Bacteroides xylanisolvens*^6^, the genera *Enterobacteriaceae* and *Bacillus*^52^, the genera *Blautia* and *Lachnospira*^6^, and the genus *Bifidobacterium*^6,7,12–14,52^.

## Discussion

We apply both the Tweedie distribution, for modeling gene and species abundances in metagenomic data, and sparse CCA to the challenge of identifying correlations between overall host genetics and the composition of the gut microbiome or its composite functions. Common species or gene families will tend to have a higher mean across samples, while also having larger variation, and a lower probability of being *0* (which here can mean either missing in the sample or not measured) in any one sample. Conversely, a rare gene function or low-abundance bacterium, may have a low mean, high variance, and a high probability of being *0* in many samples. While the Tweedie distribution is a superior fit for both metagenomic gene family and species abundance data, it has not yet been widely applied to genomics. Our use of the Tweedie distributions makes possible the modelling of the compositionality corrected outcomes. After correcting for the compositionality using a modified CLR transformation, the read counts become arbitrary non-negative numbers rather than counts. Therefore, count based distributions such as the Poisson and Negative Binomial cannot be used for modelling in our scenario. In contrast, the Tweedie distribution both has the correct support (non-negative numbers), has the ideal mean–variance relationship (illustrated in Fig. 2A,B), models the probability of zeroes, and models the probability of zeroes appropriately by accounting for low values having a higher probability of zero (illustrated in Fig. 2C,D). Additionally, if we remove all zeros from the metagenomic gene abundance data, Tweedie still captures the mean-to-variance relationship better than other distributions, showing the value of Tweedie beyond the problem of zero-inflation (Supplemental Fig. S1).

Our choice of penalties for sCCA have known features that affect the interpretation of results: (1) group lasso, which we applied to the human variants, enables us to select SNPs as a whole^45^ and (2) elastic net, which we applied to the metagenomic features, will tend to select a larger set of correlated features within a sample if genes or species are correlated with each other^46^. When considering metagenomic gene family abundances, we can intuitively imagine that once one gene from a pathway is selected, the model will select the whole group of correlated genes from the same pathway. This behavior of elastic net variable selection makes it ideal for analyzing gene abundances. sCCA with elastic net searches for the overall effect of many smaller, combined effects, which while appropriate for genes that can be grouped into functional pathways, may also capture biologically relevant relationships between microbiota and their host. For example, co-occurrence of bacterial species in the gut microbiomes may capture how multiple species can work together to perform a function such as metabolic cross feeding that results in the production of a biologically important metabolite.

From our two analyses on microbial gene family abundances, across the first two components, we find that 339/12,813 gene families were found to be associated with host genetics, or 2.64%. When we apply this method to investigate microbial taxa abundances, the first two components suggest that the abundances of 485, or 74.04%, of species in this analysis are associated with host genetics. These represent the results of sCCA when two canonical components have been generated. It is possible to extract additional components until all features have been selected. In this situation, each canonical component would represent a new dimension of the data.

While sCCA identifies a small number of known gene-microbiome relationships, it expands the number of broadly associated human SNPs with microbiome composition and function. Our results show that human genes known to be associated with human diseases such as type 2 diabetes and schizophrenia are associated with components of the gut microbiome and are significantly enriched in our data. These results may provide new routes of study for identifying links between the gut microbiome and disease risk. We identify many microbial species that are known components of a healthy gut microbiome and expand upon the list of species that may be heritable. We also identify abundant crAssphage, which are the most abundant bacteriophage in the human gut, as being associated with human genetics. CrAssphage are a gut-associated bacteriophage which is broadly associated with primates^54^. In addition, while most members of the gut virome are unique between twins and family members, crAssphage genomes tend to be nearly identical between mothers and infants, indicating a heritable component^54^.

We identified many bacterial functions and pathways that are associated with host genetics. Secretion systems, particularly type VI secretion systems, are used by bacteria to interact with each other in the human gut microbiome in antagonistic and cooperative ways. For instance, *Bacteroidales* species have been shown to transfer type VI secretion system loci between species and families within the order^55^. Pathways related to transportation are also associated with host genetics in this population. Translocases, and the two-component system may be involved in host-microbe, or microbe-microbe interactions in the gut microbiome. There is evidence for the role of mitochondrial translocases in the host-microbe crosstalk that may be important in colorectal cancer or inflammatory bowel diseases^56^. Additionally, peptidoglycans may be important signaling molecules for interactions between the gut microbiome and the nervous system^57^.

A drawback of our penalized CCA method is that by using regularization methods in the first part of the method, we cannot readily interpret the results as we would for a traditional CCA. For example, the coefficients for the metagenomic species and gene abundances cannot be ranked since the standard errors are unknown and the shrinkage from the penalization may have had a different effect across features. That is, properly controlling for variable selection makes inference on the sCCA coefficients more difficult than it is classically. Additionally, the results have a specific interpretation that the associated SNPs and metagenomic abundances share a common latent factor^58^. In other words, there is no implication of directionality between the SNPs and metagenomic gene abundances.

Given that this method requires paired metagenomic (shotgun metagenomic sequencing or 16S rRNA sequencing) and host genome data, we are still limited by what is publicly available. Our analyses are performed on a small, healthy cohort of women from the United Kingdom. We do not expect large differences in gut composition/function within a mostly healthy cohort, so effect sizes are small. A larger sample from more diverse geographic and cultural backgrounds would enhance our ability to identify novel associations between the human gut microbiome and human genetics. Despite the usual limitations of a small sample size, we were still able to uncover previously known associations as well as novel ones. It would be interesting to perform our method on a large disease cohort where strong differences between disease and control microbiomes are expected and may provide more context for disease-associated mechanisms. Additionally, there are many known SNPs involved in microbiome-related disorders and larger microbiome differences between disease and control samples would make it more likely to find additional SNPs that correlate with these overall shifts.

The methods presented in this paper are applicable to other studies, and many of our methodological adjustments are flexible. For instance, we constructed a custom gene catalog from the metagenomes, but using available software such as HUMAnN3^59^ to estimate gene or species abundances would give similar results. Additionally, we applied our methods to a twin’s dataset, but through our Tweedie GLMM, we treated them as unrelated individuals. With a cohort of unrelated individuals, the mixed effect GLMM would simply need to be modified to a standard GLM with Tweedie. In terms of sCCA itself, the flexible part is the choice of regularization method. We used group lasso to select SNPs but depending on the given coding scheme for the SNPs, lasso could just as easily be swapped into the method.

## Methods

### Subject details and data

The study involved metagenomic sequencing from a subset of individuals from the TwinsUK Project at King’s College London as reported previously^33,34^. Deidentified metagenomic data were processed to remove adapter sequence, low quality, duplicate, and human reads^60–62^. High quality reads were assembled into contigs using MetaSpades v3.13.1^63^. All work involving human subjects was approved by the Cornell University IRB (Protocol ID 1108002388) and all methods were performed in accordance with relevant guidelines and regulations. Informed consent was obtained from all participants.

### Gene Catalog construction

Prodigal v2.6.3^64^ was used to predict proteins on the assembled contigs. Duplicate proteins were removed using vsearch v2.15.0^65^ and sequence redundancy was further removed by CD-hit v4.8.1^66^ (90% identity) leading to a catalog of 12,563,449 nonredundant proteins. DNA sequences for the clustered proteins were used to form the final gene catalog for alignments.

### Metagenomic gene family abundance calculation

Metagenomic reads were aligned to the gene catalog using BWA mem v0.7.17 (-a -bwtsw -t 4)^67^. The output was filtered to retain primary alignments and reads aligning with at least 90% identity. Next, genes were removed if less than 80% of the gene was covered. Abundances were then normalized using a modified RPKM approach according to the gene length and the geometric mean of abundances per sample to account for the compositionality of the data. Genes were annotated using KEGG^68^ orthologs (downloaded 2016) and abundances were aggregated by KO into functional gene families leading to a final abundance table with 12,645 microbial gene family abundances.

### Genotype data

Human genotyping data are available for 240 individuals from an Illumina HumanHap300 Bead Chip or Illumina HumanHap610 Quad Chip and imputed using IMPUTE version 2^34,69^. One individual from each monozygotic twin pair was genotyped and the data were duplicated for the full pair. SNP data were filtered to remove missing data, rare alleles, and loci violating HWE or in high LD (> 80%) with one another (Plink v1.9 –geno 0 –maf 0.10 –indep-pairwise 50 10 0.8 –hwe 0.001)^70^. Variants are coded for additive and dominance effects such that the additive component is {1, 0, -1} for the number of alleles, and the dominance effect is coded as {-1, 1, -1} such that these effects are orthogonal to the additive effects.

### Microbial taxa abundances

First, metagenomic reads were annotated to the species level using Kraken2 (v2.1.0, –confidence 0.1)^71^. The libraries for Kraken2 were downloaded individually (bacteria, archaea, plasmid, UniVec_Core, viral, and human), then the standard database was built (kraken2-build –standard). Then abundances were estimated using Bracken with a minimum of 10 reads required for classification at the species level (v2.0, -r 100, -l S, -t 10)^72^. Species abundances were normalized by the geometric mean per sample and species present in at least 10% of the population were retained. A total of 655 species were analyzed.

### Statistical analyses

Prior to association testing, a Tweedie generalized linear mixed model was performed in R^44^ using the lme4 (v1.1.21) and statmod (v1.4.35) packages to extract residuals from the gene, species, and SNP data. The models include fixed terms for the individual’s age at sampling, their BMI, the shipment number of their sample, and a random effect for their twin status (either monozygotic or dizygotic) to remove the effect of a person’s correlation with their own twin otherwise known as relatedness. The use of the twins’ zygosity and genetic differences between them is a topic for another paper. Further, we included ten principal components from the human SNPs in the models to remove any effects of ancestry from the data, where the principal components were fit after removing all co-twins (i.e., only one individual from each twin pair is used to perform PCA).

### Sparse canonical correlation analysis, sCCA

We use a multivariate method to associate human variants and microbial gene abundances called sparse canonical correlation analysis, or sCCA. Similar to principal components analysis, CCA projects observations into lower dimensional space, but has the added benefit of comparing across tables. CCA aims to identify the linear combination of two tables that maximizes the correlation between the two. Traditional CCA requires low-dimensional data where the sample size is larger than the number of features, which is not the case for modern genomic datasets. In order to apply CCA to high-dimensional data, we apply a penalized variant of CCA, or sCCA, that first performs variable selection to reduce the number of features to be fit. For the genes and species input data, we apply elastic net regularization, which combines the *l*_*1*_ and *l*_*2*_ penalties from lasso and ridge regression methods, that is:

$$\mathop {{\text{argmin}}}\limits_{{\alpha , \beta : \left\| {X\beta } \right\|_{2} = \left\| {Y\alpha } \right\|_{2} = 1 }} \left\| {X\beta - Y\alpha } \right\|_{2}^{2} + \lambda_{1} pen_{1} \left( \beta \right) + \lambda_{2} pen_{2} \left( \alpha \right)$$

We apply group lasso to the variants in order to select the entire variant, however variations of this method could use different variant coding or different regularization methods. The first stage of this method is to perform tuning parameter optimization to select the *l*_*1*_ and *l*_*2*_ penalty via cross validation. Twelve pairs of penalty parameters are tested via cross validation. Once the penalties are selected, we perform sparse CCA with the gene or species residuals, the variant residuals, and the selected penalties. The output is a list of all inputs (genes or species, and variants) with coefficients that are either zero or nonzero. Features with a nonzero coefficient was selected by sparse CCA as having the maximum correlation between the two input tables. We fit the sCCA through block coordinate descent by iterating regression on X and on Y. To extract additional relationships using sCCA, we use the part of the data unrelated to the first component in a process known as matrix deflation. In other words, we look at the residuals of the SNP matrix after running a regression with the first set of alpha values to remove the effect of the first component.

Statistical analyses and association tests were performed in R. Code adapted from glmnet (v4.1–1), gglasso (v1.4), and lme4 (v1.1.21), to perform penalized CCA with elastic net and group lasso. The new reduced set of gene family abundances and human variants are then fit by CCA for each component requested.

### sCCA results analyses

BiomaRt^73^ was used to annotate the human SNPs using HG19. FUMA GWAS was used to annotate the selected SNPs by their gene-disease associations and functions (GO terms) and performed enrichment analyses to identify GWAS associations for human SNPs and genes^47^.

Using the KEGG API, pathway information was linked to the microbial gene family abundances selected by CCA. Fisher’s exact test with false discovery rate correction performed in R for pathway enrichment test.

## Supplementary Information

Supplementary Information 1.

Supplementary Table S1.

Supplementary Table S2.

Supplementary Table S3.

## Data Availability

The accession number for metagenomic shotgun sequencing data for all 250 samples after removal of human sequences reported in this paper is European Bioinformatic Institute (EBI): ERP010708.

## Code availability

All code and scripts used to perform the analyses in this paper are available at: https://github.com/fnew/snp_metagenomes.
