# Supplementary material for: Collective effects of human genomic variation on microbiome function
Source: Sci Rep. 2022 Mar 9;12:3839. doi: 10.1038/s41598-022-07632-3 (PMC8907173; doi:10.1038/s41598-022-07632-3)
Supplement: Supplementary file 1 — Supplementary Information 1. [file 41598_2022_7632_MOESM1_ESM.pdf]

Supplemental Information

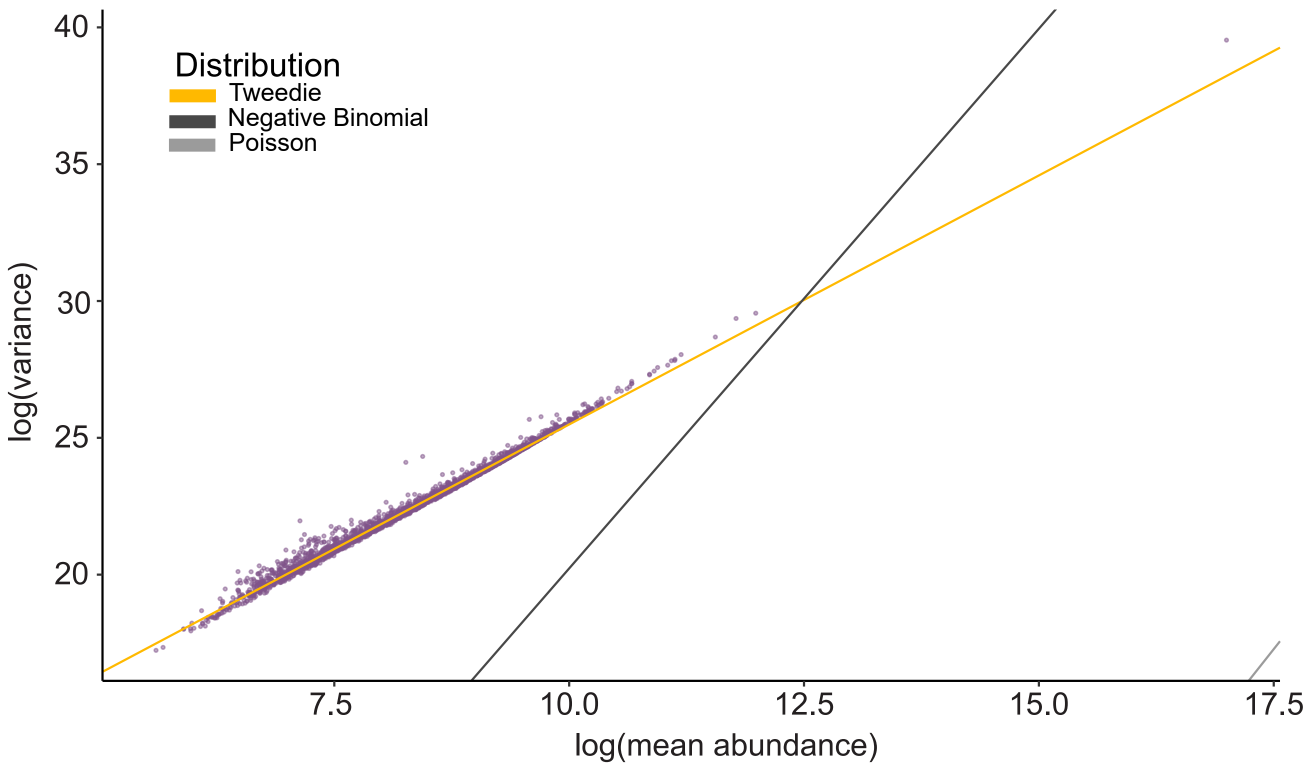

Supplemental Figure S1 (above). Tweedie distribution captures the mean-to-variance relationship of the metagenomic abundances even when all zeros have been removed from the data. Zeros were removed from the data and the log mean abundances were plotted against the log variance of the abundances. The yellow, dark grey, and light grey line represents the best fit for Tweedie distribution, negative binomial, and the Poisson distributions, respectively. Figure created in R v4.1.2 (https://cran.r-project.org)[44].

Supplemental Table S1. sCCA Results. Sheet A contains the table of contents. Sheet B contains the output of sCCA for the human SNPs for each of the four analyses performed in the paper. Sheet C contains the results from sCCA for the microbial gene family functions (KEGG orthologies) along with their prevalence in the dataset. Sheet D contains the results from sCCA for the microbial species abundances as well as their prevalence in the dataset.

Supplemental Table S2. Annotations and enrichment tests of the SNPs selected by sCCA. Sheet A contains a table of contents. Sheets B, D, F, and H contain the annotations of the SNPs selected by sCCA from each analysis. Sheets C, E, G, and I contain the enrichment tests performed on the SNPs selected by sCCA from each analysis.

Supplemental Table S3. Enrichment tests of the human genes associated with the SNPs selected by sCCA. Sheet A contains the table of contents for the spreadsheet. Sheets B-E contain the enrichment tests on the human genes associated with the SNPs selected by each sCCA analysis.
